# Association of the etiology and peak level of markedly elevated aminotransferases with mortality: a multicenter study

**DOI:** 10.1097/HC9.0000000000000149

**Published:** 2023-04-26

**Authors:** Ji Yoon Kwak, Hyun-gyu Kim, Ji Hee Han, Hankyu Jeon, Ra Ri Cha, Sang Soo Lee

**Affiliations:** 1 Department of Internal Medicine, Gyeongsang National University School of Medicine and Gyeongsang National University Changwon Hospital, Changwon, Republic of Korea; 2 Department of Internal Medicine, Gyeongsang National University School of Medicine and Gyeongsang National University Hospital, Jinju, Republic of Korea; 3 Institute of Health Sciences, Gyeongsang National University, Jinju, Republic of Korea

## Abstract

Markedly elevated aminotransferase levels are commonly encountered among hospitalized patients. However, data regarding the trajectory of enzyme elevation and disease-specific prognosis are limited.

**Methods:**

This study included 3237 patients with at least one episode of aspartate aminotransferase or alanine aminotransferase level being higher than 400 U/L between January 2010 and December 2019 at 2 centers. Patients were classified into 5 groups comprising 13 diseases according to etiology. Factors associated with 30-day mortality were evaluated using a logistic regression analysis.

**Results:**

The most common disease leading to markedly elevated aminotransferase level was ischemic hepatitis (33.7%), followed by pancreatobiliary disease (19.9%), DILI (12.0%), malignancy (10.8%), and viral hepatitis (7.0%). The 30-day all-cause mortality rate was 21.6%. The mortality rate for patients from the pancreatobiliary, hepatocellular, extrahepatic, malignancy, and ischemic hepatitis groups was 1.7%, 3.2%, 13.8%, 39.9%, and 44.2%, respectively. Age, etiology, and peak aminotransferase levels were independently associated with 30-day mortality.

**Conclusions:**

In patients with markedly elevated liver enzymes, the etiology and peak AST level are significantly associated with mortality.

## INTRODUCTION

Elevated serum aspartate aminotransferase (AST) and alanine aminotransferase (ALT) levels are a hallmark of hepatocellular injury. Both AST and ALT are highly represented in the liver. AST is also diffusely found in the heart, skeletal muscle, kidneys, brain, pancreas, lungs, leukocytes, and erythrocytes. In contrast, ALT has low concentrations in other tissues. Thus, elevated ALT is a more specific marker than AST for hepatocellular injury.^1^,^2^

Marked elevation of serum aminotransferase levels is frequently encountered in clinical practice. Recently, several studies on the etiology of the marked elevation of serum aminotransferase have been undertaken.^3^–^7^ The differential diagnosis of a markedly elevated aminotransferase level is typically ischemic hepatitis, followed by DILI, viral hepatitis, and biliary obstruction. The overall mortality rates in patients with markedly elevated aminotransferase levels are relatively high (15%–55%),^3^,^5^,^6^,^8^–^10^ which may vary depending on the etiology of hepatic injury. Ischemic hepatitis is associated with a high mortality rate (~50%) and usually occurs in the intensive care unit setting.^11^–^13^ Hepatocellular injury (such as viral hepatitis and DILI) has an extremely good prognosis, and these patients can almost always be managed in general hospital wards or in an outpatient setting.^4^–^6^,^14^ Biliary obstruction associated with benign pancreatobiliary disease has emerged as a major cause of markedly elevated serum aminotransferase levels in recent studies,^5^,^7^,^10^,^15^ and these patients have a low mortality rate of <3%.^5^,^10^,^16^

The mortality rates are the highest in patients with ischemic hepatitis and hepatobiliary malignancy (37%–75%),^3^,^5^,^6^,^8^,^10^ whereas they are the lowest in those with benign pancreatobiliary disease, acute viral hepatitis, and DILI (0–7%).^4^–^6^

Therefore, we conducted a large multicenter observational study of all patients with markedly elevated aminotransferase levels (>400 U/L) to determine their common etiologies and to investigate the factors associated with mortality.

## METHODS

### Study population

**FIGURE 1:**
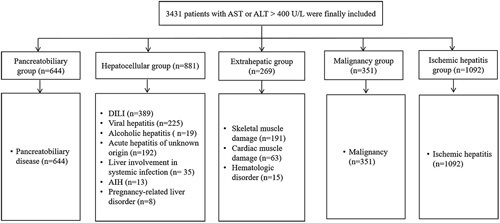
The enrolled patients were divided into 5 groups according to etiology. Abbreviations: AIH, autoimmune hepatitis; ALT, alanine aminotransferase; AST, aspartate aminotransferase.

To capture all patients with diverse etiologies, a retrospective electronic medical record review was performed on 4542 consecutive patients with aminotransferase levels >400 U/L at 2 centers between January 2010 and December 2019. The exclusion criteria were as follows (Supplemental Figure S1, http://links.lww.com/HC9/A266): (1) age <18 years (n=74); (2) failure to classify the etiology due to lack of available data (n=746); (3) liver surgery or liver trauma (n=207); (4) loss to follow-up within 30 days (n=234); and (5) lack of consistent liver function test data (n=44). The remaining 3237 patients with at least one episode of AST or ALT level being >400 U/L were included (Figure 1). For patients with multiple episodes of markedly elevated aminotransferase levels (>400 U/L) during the study period, only the first episode of aminotransferase levels >400 U/L from each patient was considered. Age, sex, underlying diseases, including liver cirrhosis, diabetes, heart failure, and end-stage renal disease, presence of hepatic decompensation, infection, initial and peak laboratory data, and clinical outcomes were extracted from the electronic medical records. The study protocol was designed in accordance with the 1964 Declaration of Helsinki. The institutional review boards of the participating hospitals approved this study and waived the requirement to obtain informed consent owing to the retrospective design.

### Study design and classification

Electronic medical charts were reviewed to determine the primary cause of elevated aminotransferase levels and 30-day mortality according to the etiological group. The index date for analysis was defined as the first date on which the aminotransferase level was observed to be >400 U/L. After enrolment, all patients underwent laboratory and imaging investigations to determine the primary cause of the marked aminotransferase elevation. Peak AST and ALT levels were defined as the highest AST and ALT levels reached within 30 days after the index date.

Patients with markedly elevated aminotransferase levels, defined as at least one episode of AST or ALT level being >400 U/L, were classified into 5 groups comprising 13 diseases according to etiology by 2 experienced hepatologists (Figure 1): pancreatobiliary group, including pancreatobiliary disease^3^; hepatocellular group, including DILI,^17^ viral hepatitis, alcohol-associated hepatitis,^18^ acute hepatitis of unknown origin, liver involvement in systemic infection,^19^ autoimmune hepatitis,^20^ and pregnancy-related liver disorder^21^; extrahepatic group, including skeletal muscle damage,^22^–^24^ cardiac muscle damage,^25^ and hematologic disorder^1^,^26^; malignancy group, including malignancy; and ischemic hepatitis group, including ischemic hepatitis.^27^ Each disease according to its etiology is defined in Supplemental Table S1 (http://links.lww.com/HC9/A270). In cases with more than one etiology, the etiology that had the most significant effect on the elevation of aminotransferase level was selected based on the judgment of experienced hepatologists. Information on all-cause mortality for all enrolled patients according to the etiological group was recorded.

Serial AST, ALT, and bilirubin levels were measured during the study period. After the first date of diagnosis of aminotransferase levels >400 U/L, all patients underwent liver function tests at least every 1–2 days. Three grades were used for classifying the severity of marked elevation of serum aminotransferase levels according to AST/ALT levels: (1) AST/ALT levels <1000 U/L (ie, 400 U/L≤AST/ALT<1000 U/L), (2) AST/ALT levels <3000 U/L (ie, 1000 U/L≤AST/ALT<3000 U/L), and (3) AST/ALT levels ≥3000 U/L. Hepatic decompensation was defined as the presence of hepatic decompensated events, including hepatic encephalopathy, ascites, or documented gastroesophageal variceal bleeding. Heart failure was defined as either a history of heart failure through chart review or objective findings of impaired left ventricular and/or right ventricular function noted on echocardiogram at enrolment.

### Statistics

PASW software (version 18; SPSS Inc., Chicago, IL) was used to process the data. Categorical data were expressed as number (%), and continuous data were expressed as mean (±SD). For categorical variables, comparisons of between-group differences were performed using the χ^2^ test or Fisher exact test. For continuous variables, Student *t* test and 1-way ANOVA were used, as appropriate. Logistic regression analysis was performed to identify independent predictors of 30-day mortality. Variables with a significance level of *p* < 0.05 by univariate logistic regression analysis were subjected to multivariate analysis. The results of the logistic regression model are reported as OR with 95% CIs. Kaplan–Meier survival curves until 30 days with log-rank tests were used to compare the all-cause mortality between groups. All statistical tests were performed using 2-sided tests, with a significance level of 0.05.

## RESULTS

### Baseline characteristics

The overall population of the study comprised 1936 (59.8%) men and 1301 (40.2%) women, with a mean±SD age of 60.2±16.9 years (Table 1). Among the 3237 patients with markedly elevated aminotransferase levels, 14.7% (n=476) had liver cirrhosis, 19.6% (n=634) had diabetes, 18.1% (n=587) had heart failure, and 2.3% (n=73) had end-stage renal disease. The mean AST and ALT levels at enrolment were 1076.1±1411.2 and 689.3±925.3, respectively. The mean peak AST and ALT levels were 1583.6±2703.2 and 893.8±1557.1 U/L, respectively.

**TABLE 1:** Clinical and laboratory characteristics of patients according to the etiological group

Characteristics	Pancreatobiliary group	Hepatocellular group	Extrahepatic group	Malignancy group	Ischemic hepatitis group	Total
No.	644 (19.9)	881 (27.2)	269 (8.3)	351 (10.8)	1092 (33.7)	3237
Age, y	66.2±14.9	50.3±16.3	53.9±18.6	61.8±11.8	65.7±15.3	60.2±16.9
Male sex	308 (47.8)	489 (55.5)	196 (72.9)	265 (75.5)	678 (62.1)	1936 (59.8)
Liver cirrhosis	12 (1.9)	73 (8.3)	30 (11.2)	205 (58.4)	156 (14.3)	476 (14.7)
Diabetes	115 (17.9)	109 (12.4)	39 (14.6)	67 (19.1)	304 (27.8)	634 (19.6)
Heart failure	17 (2.6)	16 (1.8)	44 (16.4)	4 (1.1)	506 (46.3)	587 (18.1)
ESRD	8 (1.2)	7 (0.8)	2 (0.7)	2 (0.6)	54 (4.9)	73 (2.3)
Hepatic decompensation	33 (5.1)	138 (15.7)	18 (6.7)	182 (51.9)	170 (15.6)	541 (16.7)
Infection	51 (7.9)	37 (4.2)	43 (16.0)	58 (16.5)	312 (28.6)	501 (15.5)
Admission at ICU	20 (3.1)	38 (4.3)	65 (24.2)	17 (4.8)	547 (52.6)	714 (22.1)
Initial values						
AST, U/L	725.2±469.8	926.7±1208.0	879.2±812.1	943.8±1307.0	1494.4±1899.7	1076.1±1411.2
ALT, U/L	525.5±352.3	958.6±1299.8	315.0±364.2	418.5±464.6	748.0±930.5	689.3±925.3
Albumin, g/dL	3.8±0.6	3.8±0.6	3.5±0.8	3.0±0.7	2.8±0.7	3.3±0.8
Bilirubin, mg/dL	3.8±3.6	4.3±5.8	1.8±2.7	6.3±7.2	2.5±3.4	3.6±4.8
ALP, U/L	253.0±224.2	170.7±141.7	107.6±117.7	370.0±374.0	139.2±149.9	192.8±212.5
LDH, U/L^a^	560.2±400.1	679.1±908.8	1514.0±1575.8	1233.6±2135.7	1739.0±3190.0	1177.7±2212.7
Creatinine, mg/dL	0.97±0.82	0.90±0.81	1.43±1.27	1.26±1.00	1.91±1.59	1.34±1.26
PT-INR^a^	1.10±0.26	1.24±0.68	1.33±0.72	1.53±0.98	2.03±1.58	1.52±1.13
Peak values						
AST, U/L	776.4±646.8	1041.4±1401.1	1321.9±1906.4	1404.5±2140.5	2619.0±3969.8	1583.6±2703.2
ALT, U/L	565.8±365.7	998.9±1035.1	556.7±1027.2	554.3±828.4	1194.7±2353.9	893.8±1557.1
Bilirubin, mg/dL	4.9±4.5	5.8±7.6	3.0±5.1	11.2±11.3	4.5±6.4	5.5±7.3

Data are presented as the mean ± SD for continuous data and number (%) for categorical data.

^a^ Data are missing for some patients.

Abbreviations: ALP, alkaline phosphatase; ALT, alanine aminotransferase; AST, aspartate aminotransferase; ESRD, end-stage renal disease; ICU, intensive care unit; LDH, lactate dehydrogenase; PT-INR, prothrombin time-international normalized ratio.

### Etiological groups

The 5 etiological groups and 13 diseases according to the etiology of patients with markedly elevated aminotransferase levels are shown in Figure 1. The most common disease with markedly elevated aminotransferase levels was ischemic hepatitis [33.7% (n=1092)], followed by pancreatobiliary disease [19.9% (n=644)], DILI [12.0% (n=389)], malignancy [10.8% (n=351)], and viral hepatitis [7.0% (n=225)]. The most common etiological group was ischemic hepatitis [33.7% (n=1092)], followed by hepatocellular [27.2% (n=881)], pancreatobiliary [19.9% (n=644)], malignancy [10.8% (n=351)], and extrahepatic [8.3% (n=269)] groups. Table 1 shows the clinical and laboratory characteristics of the patients in the etiological group. Patients in the ischemic hepatitis group had significantly higher initial AST, peak AST, and peak ALT levels than did those in the other groups. However, initial ALT levels were highest in the hepatocellular group (Supplemental Figure S2, http://links.lww.com/HC9/A267). The ischemic hepatitis group included cases with heart failure [46.3% (n=506)], pre-existing end-stage renal disease [4.9% (n=54)], and infection [28.6% (n=312)], the rates of all of which were higher than those in the rest of the groups. In the malignancy group, pre-existing liver cirrhosis (58.4%) and hepatic decompensation (51.9%) were the most frequent among all groups.

### Clinical outcomes

At enrollment, we identified 71.9% (n=2326) of the patients to have initial AST levels <1000 U/L, 23.2% (n=751) to have initial AST levels <3000 U/L, and 4.9% (n=160) to have initial AST levels ≥3000 U/L. During the follow-up period, 62.0% (n=2008) of the patients had a peak AST level <1000 U/L, 27.9% (n=903) patients had a peak AST level <3000 U/L, and 10.1% (n=326) patients had a peak AST level ≥3000 U/L. Progression of AST levels occurred in 13.7% (n=318) and 9.6% (n=72) of the patients with initial AST levels of <1000 and <3000 U/L, respectively (Supplemental Figure S3A, http://links.lww.com/HC9/A268). Progression of ALT levels occurred in 8.7% (n=234) and 5.2% (n=24) of the patients with initial ALT levels of <1000 and <3000 U/L, respectively (Supplemental Figure S3B, http://links.lww.com/HC9/A269). Overall, 45.8% (n=1477) of the patients with markedly elevated aminotransferase levels developed hyperbilirubinemia (>3 mg/dL) during the study period. Of the 3237 patients, 22.1% (n=714) were admitted to the intensive care unit and 16.7% (n=541) had hepatic decompensation at enrolment. In the entire group, the 7-day and 30-day mortality rates were 16.2% and 21.6%, respectively.

### Etiology and mortality

**FIGURE 2:**
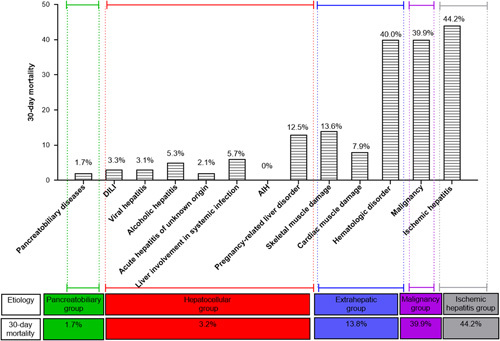
Mortality within 30 days according to etiology. Abbreviation: AIH, autoimmune hepatitis.

**FIGURE 3:**
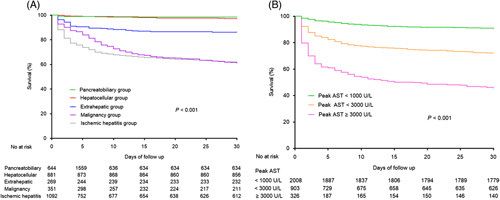
Kaplan-Meier curve for survival according to (A) etiological group and (B) peak AST level. Abbreviation: AST, aspartate aminotransferase.

The effect of etiological disease on mortality at 30 days is shown in Figure 2A. Interestingly, mortality in patients with marked elevation of serum aminotransferase levels largely depends on the etiology of liver injury. Although the 30-day mortality rate for all patients with marked elevation of serum aminotransferase levels was high, most of the deaths (623/699, 89.1%) occurred in the malignancy and ischemic hepatitis groups, consistent with the results of previous studies.^3^ The pancreatobiliary, hepatocellular, and extrahepatic groups had 30-day mortality rates of 1.7%, 3.2%, and 13.8%, respectively. Moreover, patients in the malignancy and ischemic hepatitis groups had a higher 30-day mortality rate compared with those in the extrahepatic (*p* < 0.001), pancreatobiliary, and hepatocellular groups (*p* < 0.001) (Figure 3A).

### Peak AST and ALT levels

A strong stepwise relationship was noted between peak AST levels and mortality. Patients with peak AST levels <1000 U/L (11.7%) had a lower 30-day mortality rate than did those with peak AST levels <3000 U/L (30.7%, *p* < 0.001) and ≥3000 U/L (57.4%, *p* < 0.001) (Figure 3B). The progression of AST and ALT levels from the initial levels to subsequent death is shown in Supplemental Figure S3 (http://links.lww.com/HC9/A269). Patients with increasing AST and ALT levels during injury had a higher 30-day mortality rate than did those without progression of AST and ALT levels.

### Risk factors associated with 30-day mortality

Logistic regression analysis was performed to evaluate the independent factors associated with 30-day mortality (Table 2). The pancreatobiliary group was used as a reference for the etiological groups, whereas peak AST levels <1000 IU/L and peak ALT levels <1000 IU/L, respectively, were used as the reference for peak AST and ALT levels in the logistic regression analyses. The factors associated with 30-day mortality in the univariate analysis were male sex, age, extrahepatic causes, malignancy, ischemic hepatitis, hepatic decompensation, albumin level, bilirubin level, creatinine level, prothrombin time-international normalized ratio, peak AST <3000 U/L, peak AST ≥3000 U/L, peak ALT <3000 U/L, and peak ALT ≥3000 U/L. In multivariate analysis, age (adjusted OR=1.02 per year, 95% CI=1.01–1.03), extrahepatic causes (adjusted OR=7.14, 95% CI=3.35–15.24), malignancy (adjusted OR=12.84, 95% CI=6.48–25.41), ischemic hepatitis (adjusted OR=12.27, 95% CI=6.36–23.68), hepatic decompensation (adjusted OR=2.08, 95% CI=1.50–2.90), albumin level (adjusted OR=0.31, 95% CI=0.26–0.37), bilirubin level (adjusted OR=1.04, 95% CI=1.02–1.07), creatinine level (adjusted OR=1.20, 95% CI=1.11–1.30), prothrombin time-international normalized ratio (adjusted OR=1.17, 95% CI=1.06–1.29), peak AST <3000 U/L (adjusted OR=1.93, 95% CI=1.50–2.48), peak AST ≥3000 U/L (adjusted OR=4.15, 95% CI=2.97–5.80), and peak ALT ≥3000 U/L (adjusted OR=1.90, 95% CI=1.03–3.51) were independently associated with 30-day mortality.

**TABLE 2:** Predictors of 30-day mortality in univariate and multivariate logistic regression analysis in patients with markedly elevated aminotransferase (n=3237)

	Univariate analysis		Multivariate analysis	
Variables	*p*	OR (95% CI)	*p*	OR (95% CI)
Male	0.002	1.31 (1.10–1.56)	0.340	0.89 (0.70–1.12)
Age, per y	<0.001	1.03 (1.02–1.03)	<0.001	1.02 (1.01–1.03)
Etiological group				
Pancreatobiliary group		Reference		Reference
Hepatocellular group	0.077	1.89 (0.93–3.82)	0.281	1.52 (0.71–3.23)
Extrahepatic group	<0.001	9.18 (4.61–18.29)	<0.001	7.14 (3.35–15.24)
Malignancy group	<0.001	38.18 (20.27–71.92)	<0.001	12.84 (6.48–25.41)
Ischemic hepatitis group	<0.001	45.64 (24.85–83.82)	<0.001	12.27 (6.36–23.68)
Hepatic decompensation	<0.001	4.30 (3.53–5.23)	<0.001	2.08 (1.50–2.90)
Albumin, g/dL	<0.001	0.15 (0.13–0.17)	<0.001	0.31 (0.26–0.37)
Bilirubin, mg/dL	<0.001	1.05 (1.04–1.07)	0.002	1.04 (1.02–1.07)
Creatinine, mg/dL	<0.001	1.64 (1.53–1.76)	<0.001	1.20 (1.11–1.30)
PT-INR	<0.001	2.65 (2.34–3.00)	0.002	1.17 (1.06–1.29)
Peak AST				
<1000 U/L		Reference		Reference
<3000 U/L	<0.001	3.34 (2.74–4.06)	<0.001	1.93 (1.50–2.48)
≥3000 U/L	<0.001	10.15 (7.84–13.14)	<0.001	4.15 (2.97–5.80)
Peak ALT				
<1000 U/L		Reference		Reference
<3000 U/L	<0.001	1.90 (1.56–2.32)	0.958	0.99 (0.72–1.36)
≥3000 U/L	<0.001	4.93 (3.42–7.11)	0.040	1.90 (1.03–3.51)

Abbreviations: ALT, alanine aminotransferase; AST, aspartate aminotransferase; PT-INR, prothrombin time-international normalized ratio.

### Impact of etiological group and peak AST level on mortality

**FIGURE 4:**
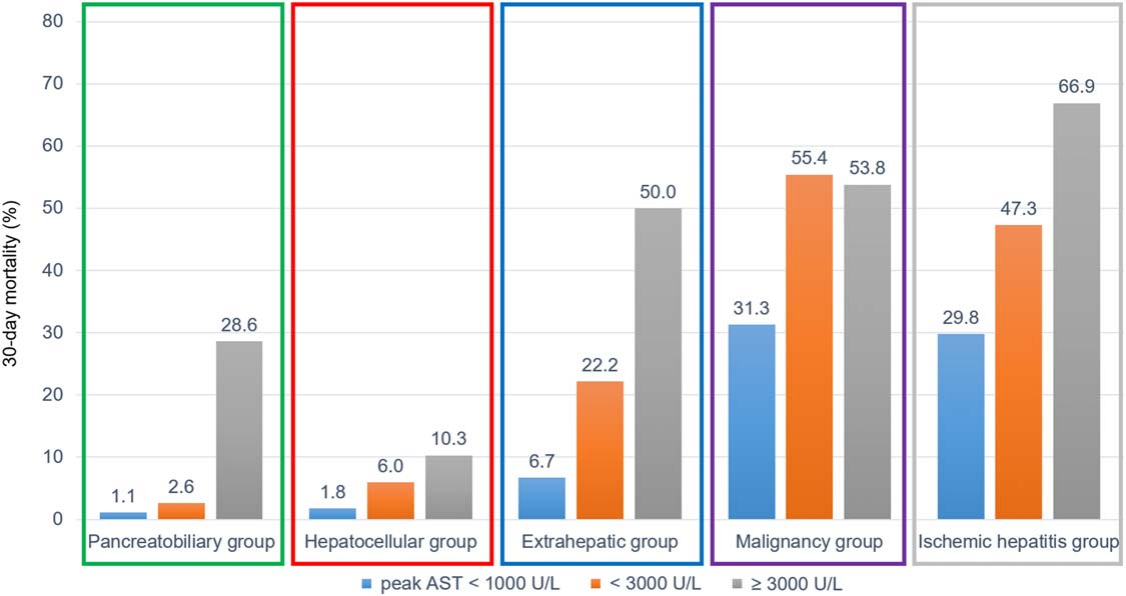
Mortality within 30 days according to peak AST level in each etiological group. Abbreviation: AST, aspartate aminotransferase.

In each etiological group, the magnitude of the peak AST level was positively correlated with 30-day mortality (Figure 4). For example, although the pancreatobiliary group had a good prognosis, the 30-day mortality rate in patients with peak AST levels≥3000 U/L was 28.6%. In the extrahepatic group, patients with peak AST levels ≥3000 U/L had a high mortality rate of up to 50%.

## DISCUSSION

In our large study of 2 centers, we identified the most frequent disease with markedly elevated aminotransferase levels (>400 U/L) as ischemic hepatitis (33.7%), followed by pancreatobiliary disease (19.9%), DILI (12.0%), malignancy (10.8%), and viral hepatitis (7.0%). The 30-day mortality was high in the entire cohort (n=699, 21.6%), but most deaths occurred in patients in the malignancy and ischemic hepatitis groups.

In the multivariate analysis, the peak AST level, rather than the peak ALT level, was a prognostic indicator in patients with markedly elevated aminotransferase levels. This is presumed to be because the AST/ALT ratio is higher in the malignancy and ischemic hepatitis groups (which have a poor prognosis) than that in the hepatocellular group (which has a good prognosis). Even in patients with acute viral hepatitis, fulminant hepatitis can occasionally lead to a high AST/ALT ratio (>2).^28^ AST is primarily present in the liver, heart, skeletal muscle, kidney, brain, and erythrocytes. Elevated AST levels are due to the cell damage caused by plasma membrane disruption and protein leakage. Circulating AST levels are influenced by several factors: direct tissue damage or apoptosis is the most common cause of viral hepatitis, drug-induced hepatotoxicity, cirrhosis, myocardial infarction, septic shock, alcohol-associated hepatitis, and skeletal muscle injury.^29^ Plasma membrane blebs that bud off from the cell membranes releasing AST are formed during ischemia reperfusion injury in the liver, skeletal muscle, and myocardium.^30^ In our study, the serum peak AST level reflects the degree and extent of injured tissues in patients with markedly elevated aminotransferase levels.

Patients with markedly elevated aminotransferase levels and high mortality rates present a challenge to physicians. In a previous population-based study from Iceland,^5^ the incidence of markedly elevated aminotransferase (>500 U/L) was 68 cases per 100,000 inhabitants per year, which suggests that events of markedly elevated aminotransferase levels are not infrequent in clinical practice. This is the largest study to investigate the etiology and prognosis of markedly elevated aminotransferase levels. Knowledge of the etiologies in patients with markedly elevated aminotransferase levels is vital for predicting the prognosis. The most common etiology of markedly elevated aminotransferase levels was ischemic hepatitis in approximately one-third of the patients, followed by pancreatobiliary disease, DILI, malignancy, and viral hepatitis. In addition, our results further elucidated the minor etiologies and their prognosis, including autoimmune hepatitis, pregnancy-related liver disorder, and extrahepatic diseases, such as cardiac muscle damage (eg, myocardial infarction and myocarditis), skeletal muscle damage (eg, rhabdomyolysis and heat stroke), and hematologic disorders (eg, hemolysis, hematoma, and hemophagocytic lymphohistiocytosis), which have not been reported in other studies. Our study showed a 30-day mortality of 21.6%, which is in line with the mortality rate of 15%–55% in previous studies with relatively small sample sizes.^3^,^5^,^6^,^8^,^9^ Previous studies revealed that mortality was the highest in patients with ischemic hepatitis and malignancy (37%–75%) 3,5,6,8,31 and the lowest in patients with pancreatobiliary and hepatocellular diseases (0–9%).^3^,^5^,^6^,^16^,^31^ These results were similar to those obtained in our study. Our multivariate analysis demonstrated that ischemic hepatitis, malignancy, and extrahepatic causes were independent factors affecting the 30-day mortality. This suggests that the etiology of markedly elevated aminotransferase levels played a major role in the clinical outcome.

In our study of 3237 patients with markedly elevated aminotransferase levels, we used an aminotransferase cutoff of 400 U/L, whereas previous studies have used cutoff levels ranging from 400 to 3000 U/L for defining markedly elevated aminotransferase levels.^3^–^9^,^31^,^32^ The all-cause mortality was 16%–22% in studies using a cutoff of >400 or 500 U/L for aminotransferase,^3^,^5^,^32^ 30%–35% in those using a cutoff of >1000 U/L,^6^,^10^ and 31%–55% in those using a cutoff of >3000 U/L.^8^,^9^,^31^ Van den Broecke et al^11^ reported that the magnitude of serum aminotransferase elevation was significantly correlated with the mortality rate in patients with ischemic hepatitis: 33.2%, 44.4%, and 55.4% for peak AST 5–10×ULN, 10–20×ULN, and >20×ULN, respectively. Therefore, we speculate that the magnitude of serum aminotransferase elevation is associated with increased mortality. We classified patients with markedly elevated aminotransferase levels into three peak AST and ALT grades. We demonstrated that the magnitude of peak AST and ALT levels was highly correlated with 30-day mortality: 11.7%, 30.7%, and 57.4% for peak AST <1000 U/L, <3000 U/L, and ≥3000 U/L and 18.0%, 29.5%, and 52.0% for peak ALT <1000 U/L, <3000 U/L, and ≥3000 U/L, respectively. Our multivariate analysis showed that peak AST <3000 U/L (adjusted OR=1.93), peak AST ≥3000 U/L (adjusted OR=4.15), and peak ALT ≥3000 U/L (adjusted OR=1.90) were independently associated with increased 30-day mortality. In addition, factors reflecting liver function, including hepatic decompensation, albumin level, bilirubin level, creatinine level, and prothrombin time-international normalized ratio, were found to be associated with 30-day mortality in multivariate analyses.

This study has a few limitations. First, its retrospective design may have limited the identification of the etiology of markedly elevated aminotransferase levels. Second, there were 780 patients for whom we failed to classify the etiology of elevated aminotransferase levels due to a lack of available data. This suggests that there may have been a bias in the population studied, especially since 618 patients with cardiopulmonary arrest on arrival were also excluded; these patients had a very poor prognosis. Despite these limitations, the strength of this study is that it is the largest study on patients with markedly elevated aminotransferase levels to date and that it shows the effect of etiology and peak AST level on mortality.

In conclusion, our study not only identified the various etiologies of patients with markedly elevated aminotransferase levels but also revealed that various etiologies have significant effects on mortality. By monitoring liver function, we demonstrated that the magnitude of the peak aminotransferase levels was significantly associated with mortality. Our observations suggest that it is important to establish the etiology of patients with markedly elevated aminotransferase levels, and that patients should be closely monitored for liver function.

## Supplementary Material

SUPPLEMENTARY MATERIAL
