# Supplementary figures and images for: Association of the etiology and peak level of markedly elevated aminotransferases with mortality: a multicenter study
Source: Hepatol Commun. 2023 Apr 26;7(5):e0149. doi: 10.1097/HC9.0000000000000149 (PMC10146537; doi:10.1097/HC9.0000000000000149)

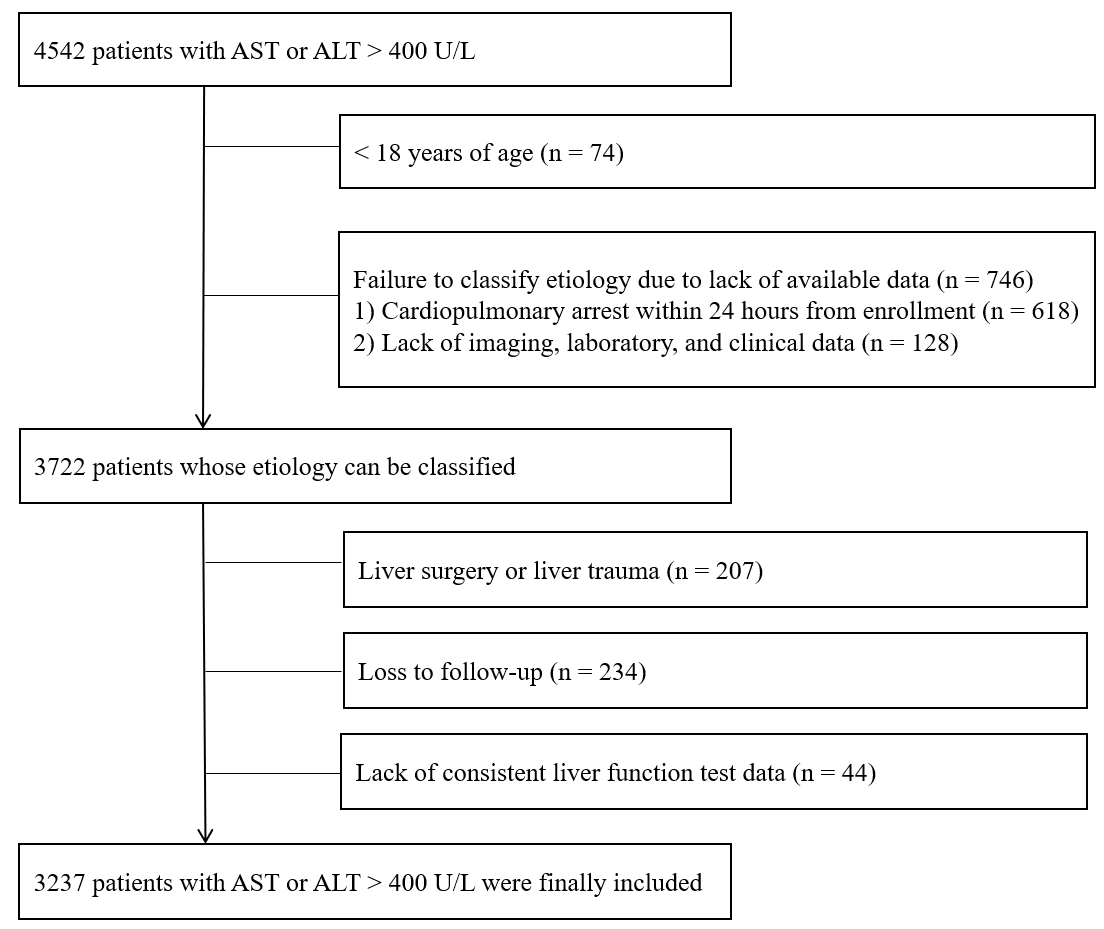

Supplement: SUPPLEMENTARY MATERIAL [file hc9-7-e0149-s001.tif]

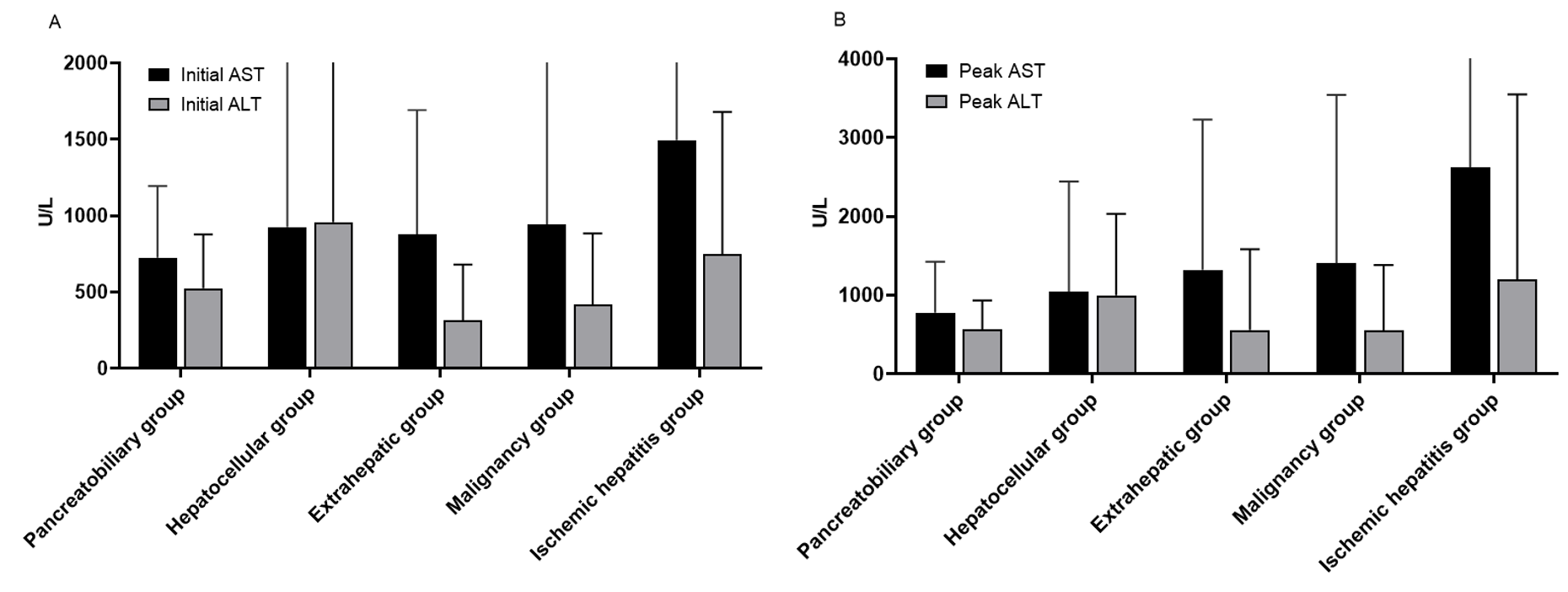

Supplement: SUPPLEMENTARY MATERIAL [file hc9-7-e0149-s002.tif]

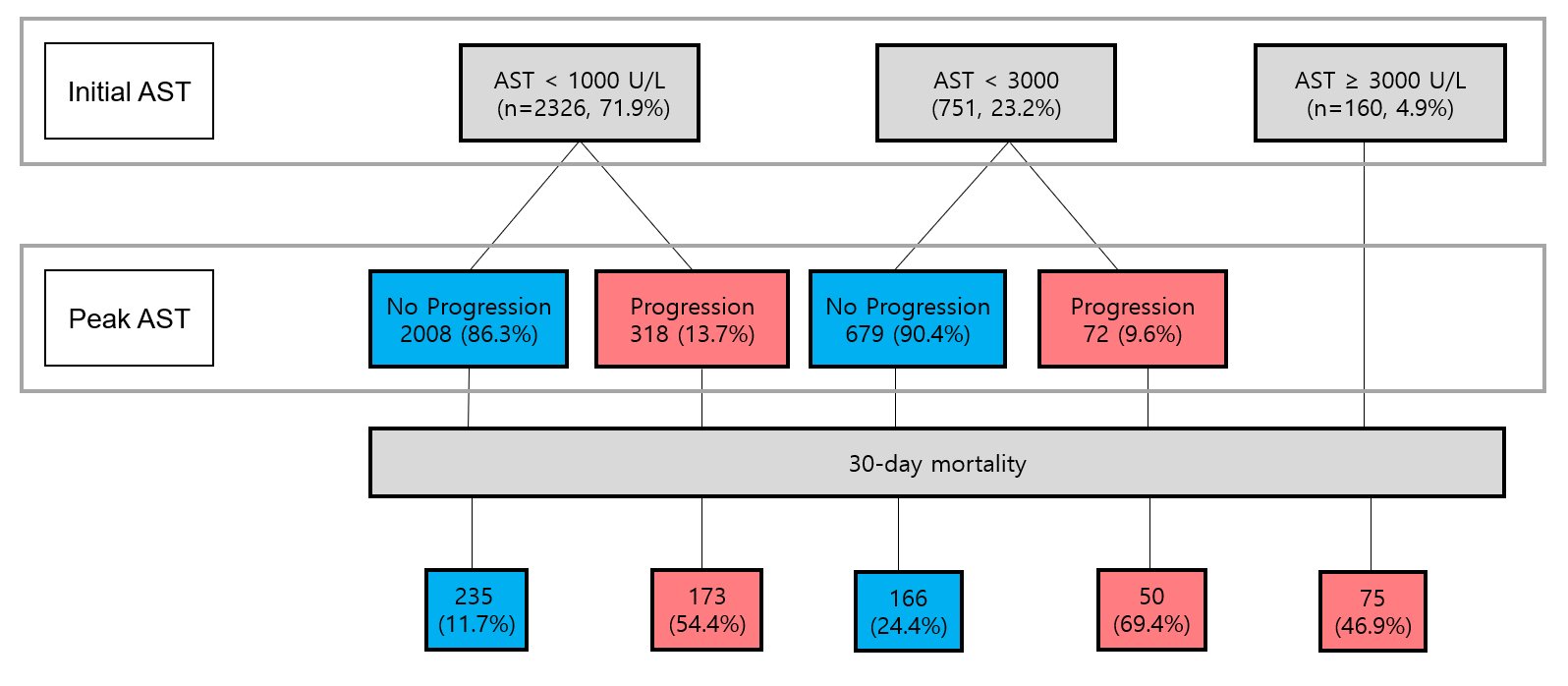

Supplement: SUPPLEMENTARY MATERIAL [file hc9-7-e0149-s003.tif]

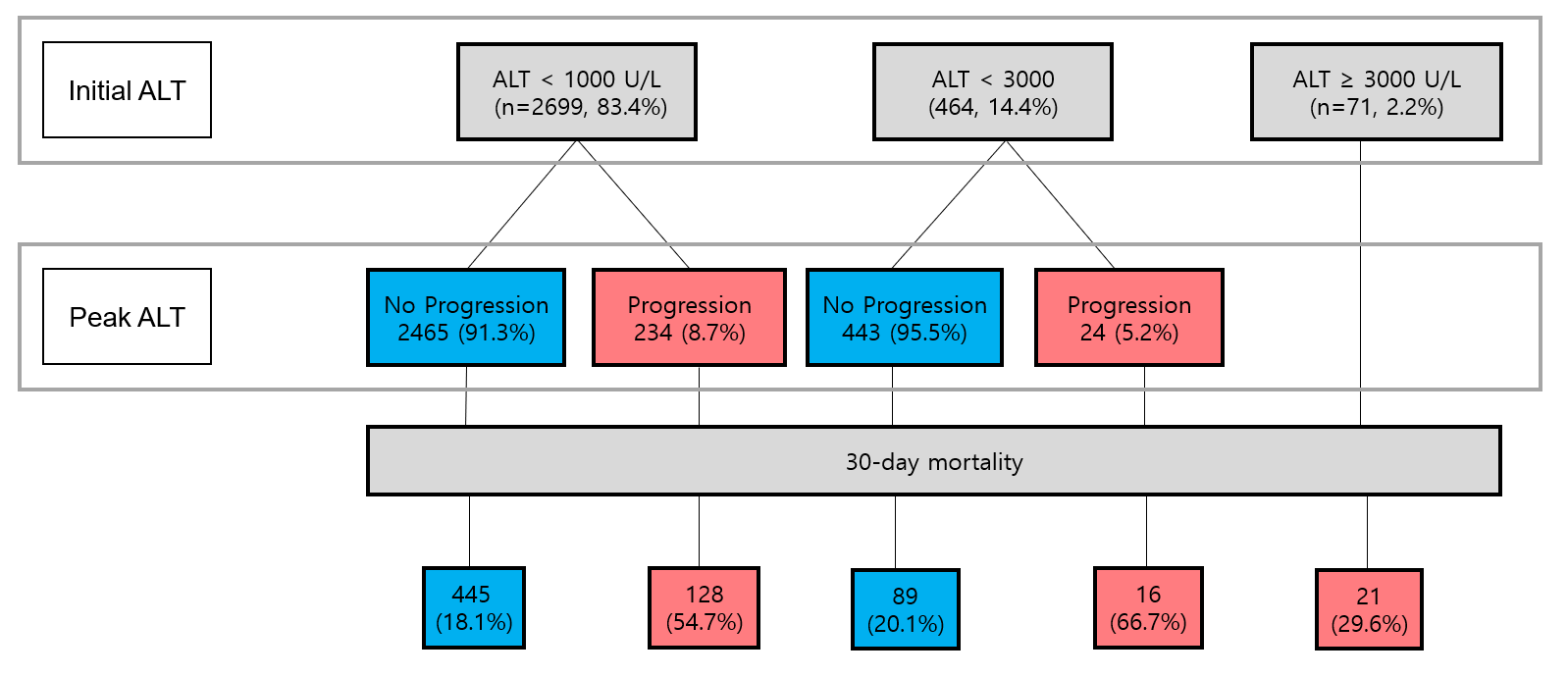

Supplement: SUPPLEMENTARY MATERIAL [file hc9-7-e0149-s004.tif]
