# Supplementary material for: Association of the etiology and peak level of markedly elevated aminotransferases with mortality: a multicenter study
Source: Hepatol Commun. 2023 Apr 26;7(5):e0149. doi: 10.1097/HC9.0000000000000149 (PMC10146537; doi:10.1097/HC9.0000000000000149)
Supplement: SUPPLEMENTARY MATERIAL [file hc9-7-e0149-s005.docx]

Supp. Table 1. Definitions for each diseases in patients with markedly elevated aminotransferase

| Group | Disease | Definition |
| --- | --- | --- |
| Pancreatobiliary group | Pancreatobiliary disease | defined as liver injury caused by benign pancreatobiliary diseases including cholecystitis, cholangitis, and pancreatitis |
| Hepatocellular group | DILI | defined as liver injury caused by various drugs, herbs, or other xenobiotics that meet the following criteria (1) appropriate temporal relationship between drug intake and the onset of liver injury, and between the drug withdrawal and clinical course of the reaction; and (2) exclusion of other potential causes of liver disease |
|  | Viral hepatitis | includes acute or chronic viral hepatitis A, B, C, D, and E. |
|  | Alcoholic hepatitis | 1) The onset of jaundice (Serum bilirubin > 3 mg/dl) within 60 days of heavy alcohol consumption (more than 50 g/day) for a minimum of 6 months  2) AST/ALT < 500 U/L  3) AST:ALT ratio > 1.5  4) No other cause of acute hepatitis (e.g., gastrointestinal hemorrhage, hypotension, rhabdomyolysis, epilepsy, ischemic liver injury, and drug-induced liver injury) |
|  | Acute hepatitis of unknown origin | A patient suspected of presenting with acute hepatitis with uncertain etiology without evidence of a viral hepatitis, systemic infection, AIH, alcoholic hepatitis, and DILI. |
|  | Liver involvement in systemic infection | Liver injury with various systemic infection that are not considered to be primarily hepatotropic viruses including Salmonella typhi infection, Mycobacterium infection, Leptospirosis, parasitic liver involvement, and non- hepatotropic viral infection (Epstein-Barr virus or cytomegalovirus) |
|  | AIH | Chronic liver disease of unknown etiology characterized by interface hepatitis on the histology of the liver biopsy, hypergammaglobulinemia, and circulating autoantibodies |
|  | Pregnancy-related liver disorder | defined as liver disease directly related to pregnancy including hemolysis-elevated liver enzymes, low platelets syndrome, hyperemesis gravidarum, acute fatty liver of pregnancy, and preeclamptic liver dysfunction |
| Extrahepatic group | Skeletal muscle damage | defined as muscle symptoms and a acute elevation in serum CK > 5000 U/L: including rhabdomyolysis and heat stroke |
|  | Cardiac muscle damage | defined as acute elevation of aminotransferase level during the first 5 day after myocardial injury without evidence of ischemic hepatitis: including acute myocardial infarction, congestive heart failure, and myocarditis |
|  | Hematologic disorder | defined as elevation of aminotransferase level associated with disorders of the blood and blood-forming organs or clinically suspected hematoma and hemolysis; Hematologic disorders included (1) malignant hematologic disorder, such as leukemia, lymphoma, and multiple myeloma; and (2) non-malignant hematologic disorder, such as paroxysmal nocturnal hemoglobinuria, thrombotic thrombocytopenic purpura, myelodysplastic syndrome, and hemophagocytic lymphohistiocytosis. |
| Malignancy group | Malignancy | defined as elevation of aminotransferase level associated with secondary to infiltration of the liver by malignant cell or with procedure, chemotherapy, and hepatic failure in patients with intrahepatic or extrahepatic malignancy |
| Ischemic hepatitis group | Ischemic hepatitis | defined as (1) sharp increase in aminotransferase > 400 U/L (2) exclusion of other causes of acute liver injury, particularly viral hepatitis, AIH, alcoholic hepatitis, or DILI, and (3) in an appropriate clinical setting of cardiac, respiratory, and circulatory failure, and septic shock |

Abbreviation: DILI, drug-induced liver injury; AIH, Autoimmune hepatitis; AST, aspartate aminotransferase; ALT, alanine aminotransferase CK, creatine kinase.
